# Muscle quality index is associated with trouble sleeping: a cross-sectional population based study

**DOI:** 10.1186/s12889-023-15411-6

**Published:** 2023-03-14

**Authors:** Yanwei You, Yuquan Chen, Qi Zhang, Ning Yan, Yi Ning, Qiang Cao

**Affiliations:** 1 Division of Sports Science and Physical Education, Tsinghua University, 100084 Beijing, China; 2 Institute of Medical Information/Medical Library, Chinese Academy of Medical Sciences & Peking Union Medical College, 100020 Beijing, China; 3 Undergraduate Department, Taishan University, 250111 Taian, China; 4 Heart Centre, Department of Cardiovascular Diseases, General Hospital of Ningxia Medical University, 750004 Yinchuan, China; 5 Department of Cardiology, The Fourth Affiliated Hospital of Harbin Medical University, 150001 Harbin, China; 6 School of Pharmacy, Macau University of Science and Technology, 999078 Macau, China

**Keywords:** Muscle quality index, Trouble sleeping, Population-based study, National Health and Nutrition Examination Survey

## Abstract

**Background:**

Trouble sleeping is one of the major health issues nowadays. Current evidence on the correlation between muscle quality and trouble sleeping is limited.

**Methods:**

A cross-sectional study design was applied and participants aged from 18 to 60 years in the National Health and Nutrition Examination Survey (NHANES) 2011–2014 was used for analysis. Muscle quality index (MQI) was quantitatively calculated as handgrip strength (HGS, kg) sum/ arm and appendicular skeletal muscle mass (ASM, kg) by using the sum of the non-dominant hand and dominant hand. Sleeping data was obtained by interviews and self-reported by individuals. The main analyses utilized weighted multivariable logistic regression models according to the complex multi-stage sampling design of NHANES. Restricted cubic spline model was applied to explore the non-linear relationship between MQI and trouble sleeping. Moreover, subgroup analyses concerning sociodemographic and lifestyle factors were conducted in this study.

**Results:**

5143 participants were finally included in. In the fully adjusted model, an increased level of MQI was significantly associated with a lower odds ratio of trouble sleeping, with OR = 0.765, 95% CI: (0.652,0.896), p = 0.011. Restricted cubic spline showed a non-linear association between MQI and trouble sleeping. However, it seemed that the prevalence of trouble sleeping decreased with increasing MQI until it reached 2.362, after which the odds ratio of trouble sleeping reached a plateau. Subgroup analyses further confirmed that the negative association between the MQI and trouble sleeping was consistent and robust across groups.

**Conclusion:**

Overall, this study revealed that MQI can be used as a reliable predictor in odds ratio of trouble sleeping. Maintaining a certain level of muscle mass would be beneficial to sleep health. However, this was a cross-sectional study, and causal inference between MQI and trouble sleeping was worthy of further exploration.

**Supplementary Information:**

The online version contains supplementary material available at 10.1186/s12889-023-15411-6.

## Introduction

Sleep time accounts for one-third of the life span in human beings while sleep health is increasingly becoming a public health concern. However, with the acceleration of social rhythm, dissatisfaction with sleep with regard to feeling difficult falling asleep or remaining asleep or waking up early was present in around one third population [1]. More and more people reported trouble sleeping and previous studies [2, 3] found that it affected 10-34% of the US population. More broadly, trouble sleeping contained obstructive sleep apnea, sleep quality complaints (sleep deprivation, sleep duration and insomnia), with a combination of other sleep problems. According to the State of the Science Conference on Manifestations and Management of Chronic Insomnia, trouble sleeping and insomnia were in the form of several complaints of disturbed sleep in adequate opportunity and circumstance for sleeping [2]. Similarly, referring to the American Academy of Sleep Medicine’s Classification of Sleep Disorders (3rd edition), it defined trouble sleeping or insomnia as a persistent difficulty with sleep initiation, duration, consolidation, or quality that occurs although acquiring adequate opportunity and circumstances for sleeping, and results in some form of daytime impairment [4]. Trouble sleeping could exist as one single problem, but also coexist with other physical or mental diseases, and long-term sleep quality decline could affect blood pressure, autonomic nervous system and cardiovascular disorders [5].

Physical fitness is proved to be related to favorable sleep patterns [6]. There is a bi-directional relationship between sleep disturbances and poor physical functioning [7]. Muscle quantity and muscle quality are two important indicators that can objectively reflect physical function. Poor sleep has been shown to impair maximal muscle strength in compound physical activities [8]. There was evidence that handgrip strength, one of the muscle quality assessment, can be considered as a predictor of mortality, functional limitations, bone mineral density, fractures, cognitive and affective disorders, and several chronic diseases [9, 10]. Current research tended to show that weak grip strength was negatively associated with poor sleep quality, sleep disturbances or impairment. One research did not detect such association, but this may be attributed to their study sample including aged adults (mean age ≥ 65) [11], while aging was independent risk factor that might lead to physical and biological changes in muscle and sleep patterns. To sum up, no consensus has been reached due to different study designs and the mixed method when assessing muscle index, and less is known about the importance of muscle quality in its relationship with trouble sleeping.

It comes to the forefront that designing a novel muscle quality index rather than using independent muscle mass or strength predicts better in the heath related outcomes. To fill this research gap, Lopes, L. et al. introduced the muscle quality index (MQI) to assess muscle quality [12]. MQI is a predictor of functional capacity, which has been previously regarded as a better indicator of muscle function compared to muscle mass or grip strength [9], which can quantify the changes in locomotion systems and predict physical functioning as well as longevity. No previous study has directly explored the relationship between MQI and trouble sleeping. Therefore, we conducted this current study and hypothesized that MQI could affect sleep health and attempted to examine whether MQI was associated with trouble sleeping in a representative nationwide population on the basis of information collected from the National Health and Nutrition Examination Survey (NHANES).

## Materials and methods

### Study population and design

This current study used data from the National Health and Nutrition Examination Survey (NHANES) cycle of 2011–2014, an ongoing program conducted by the National Center for Health Statistics in the Centers for Disease Control and Prevention (CDC) [13]. The reason for choosing the 2011–2014 period was that MQI test was only available in 2011–2012 and 2013–2014 cycles. Research procedure of NHANES was approved by the Institutional Review Board (IRB) of the National Center for Health Statistics (NCHS), with written informed consent obtained.

**Fig. 1:**
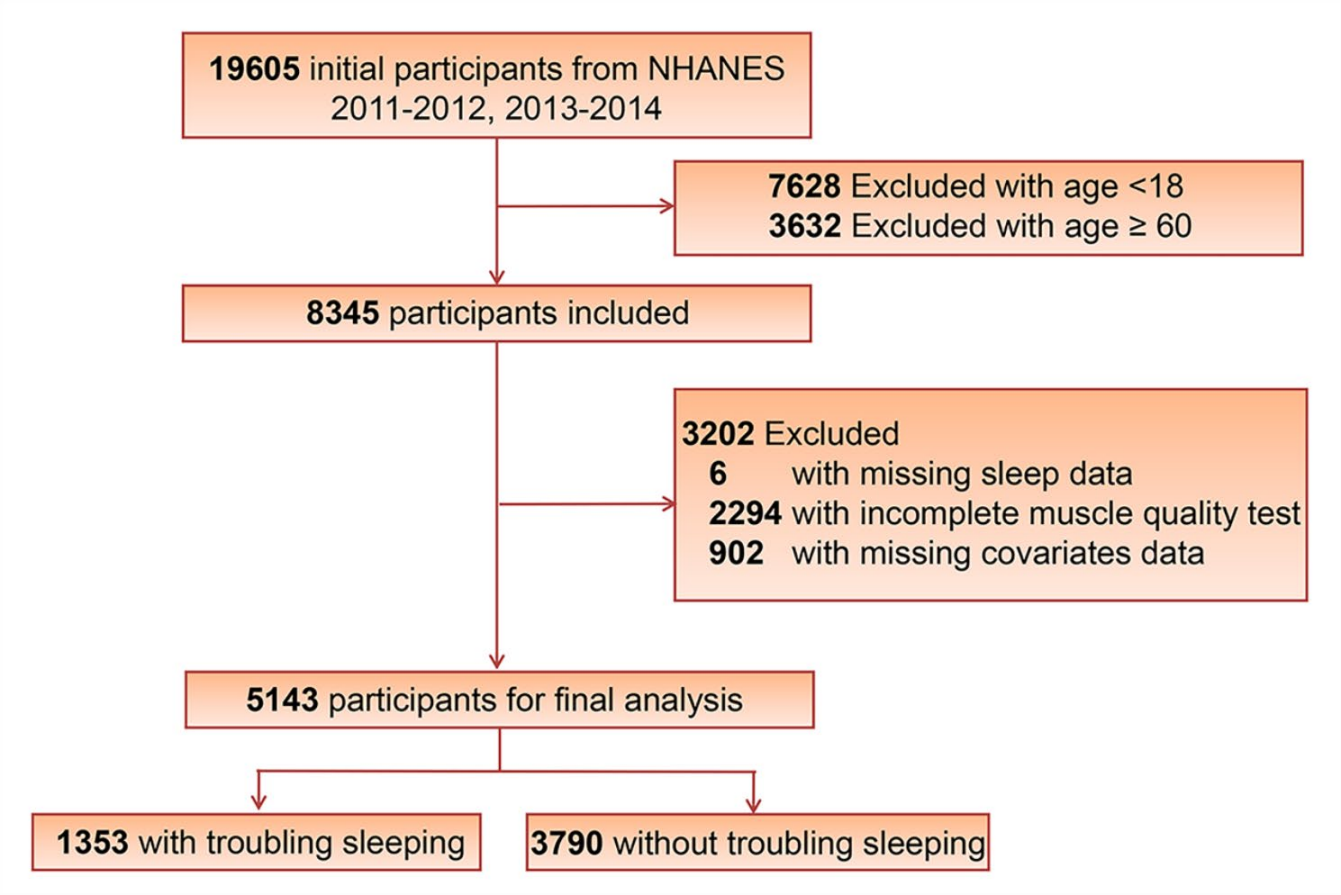
The flow chart of study population selection

A total of 19,605 participants were initially identified in these two cycles. We excluded participants below 18 (n = 7628) and over 60 (n = 3632) years of age, due to the fact that body composition examination of individuals was not available for these population. After excluding participants with missing data on trouble sleeping (n = 6), MQI test (n = 2294), and covariates (n = 902), 5143 participants were enrolled in the study (Fig. 1).

### Measurement methods

Exposure (independent) variable in this study was MQI. MQI was expressed as the ratio between combined handgrip strength (HGS) (values from the dominant and non-dominant hand) divided by the total arm and appendicular skeletal muscle (ASM) mass. Hand dynamometer was applied to measure the HGS and dual-energy X-ray absorptiometry (DXA) was applied to assess the ASM. HGS was tested by a Takei dynamometer (TKK 5401; Takei Scientific Instruments, Tokyo, Japan) and examination details has been described elsewhere [12]. To calculate the ASM, the sum of lean soft tissue from four limbs was tested by body composition, which was assessed by DXA. Details regarding DXA can be found in NHANES data documentation files and related indexes have been described in one previous publication [12].

The outcome variable in this study was trouble sleeping. Sleep trouble data was obtained through interviews and reported by individuals. Self-reported trouble sleeping was assessed by the question: “Have you ever told a doctor or other health professional that you have trouble sleeping?” The response was categorized into “Yes,“ “No,“ “Refused,“ and “Do not know”. The responds “Do not know” or “Refused” were regarded as missing responses.

### Covariate assessment

Covariate assessment contained both sociodemographic and lifestyle characteristics [14]. Sociodemographic characteristics included age, sex, race/ethnicity, education level, marital status, poverty status, and body mass index. Demographic data on age, sex, race (non-Hispanic white, non-Hispanic black, Mexican American, and other races), education (Below high school, high school, and college or above), and marital status (never married, married/living with partner, widowed/ divorced) were obtained from the interview. Family poverty income ratio was classified into three levels: low income (< 1), middle income [1,3), and high income (≥ 3)). Body mass index (measured weight in kilograms divided by measured height in meters squared) was classified into three groups (< 25, [25, 30), ≥ 30).

Lifestyle characteristics included smoke, alcohol drinking, sleep duration, recreational activity, work activity and sedentary status. Information regarding smoke and alcohol drinking status were obtained from the questionnaires of smoking cigarette and alcohol use. Sleep duration was assessed by interview and was categorized into three groups: < 7 h for short duration, [7, 9) hours for normal duration, and ≥ 9 h for long duration. The physical activity status of participants was evaluated on the basis of the Global Physical Activity Questionnaire[15], which contains questions related to daily work, leisure time, and sedentary activities. According to the World Health Organization (WHO) Guidelines on Physical Activity and Sedentary Behavior [16, 17], participants were classified as insufficient and sufficient work activity (˂ 150 and ≥ 150 min/week), insufficient and sufficient recreational activity (˂ 150 and ≥ 150 min/week), sedentary behavior (≥ 480 min/day and ˂ 480 min/day) based on the self-reported time usually spending sitting on a typical day.

### Statistical analysis

Analyses were performed by R, version 4.0.5 (R Project for Statistical Computing). Due to the complex multi-stage (incorporating sample weights, stratification, and clustering) sampling design, proper weighting procedures were applied following the NHANES analytical guidelines. After 2002, NHANES weights were calculated every 2 years, and the data for 2011–2014 covered double 2-year sampling cycles. The recalculated weights were represented as (1/2) × WTMEC2YR_11–12_ plus (1/2) × WTMEC2YR_13–14_, where WTMEC2YRs were variables from NHANES 2011–2014. For weighted characteristics description, continuous variables were presented as means ± standard error (SE), and categorical variables are presented as percentages (%). Additionally, weighted logistic regression was established to estimate odds ratios (ORs) as well as 95% confidence intervals (CIs) for the associations between MQI and trouble sleeping. Firstly, the Model 1(crude model) was analyzed with no covariate adjusted. Then, Model 2 adjusted for age, sex, and race/ethnicity. Model 3 was the fully adjusted model, which further adjusted for body mass index, marital status, sleep duration, education attainment, poverty income ratio, smoking status, alcohol drinking status, physical activity and sedentary behavior. A piecewise regression analysis on the basis of the logistic regression models was performed to determine the inflection point. Restricted cubic spline (RCS) regression [18] was used to describe the non-linear relationship. Moreover, subgroup analyses were performed to investigate whether the association was modified by sociodemographic or lifestyle characteristics in the fully adjusted model. P values < 0.05 were considered statistically significant.

## Results

Table 1 shows the basic characteristics of the 5143 participants (the weighted population was 121,083,788), of whom 1353 (26.31%) had trouble sleeping problems. The mean MQI was 3.40 for our population. Among all participants, roughly half were male (51.49%). Over half of the population was Non-Hispanic White (64.70%), and had a higher education level reaching college or above (65.94%). Compared with the participants without trouble sleeping, the participants with trouble sleeping were more likely to be older (age ≥ 44), female, non-Hispanic White, living alone, and overweight (BMI ≥ 30).

**Table 1:** Weighted characteristics of participants in the NHANES (2011–2014) by trouble sleeping

Variable	All participants	Non-trouble sleeping		Trouble sleeping		*P-value*
Age						< 0.001
< 30	26.33	29.80		16.63		
[30, 44)	33.30	34.08		31.11		
≥ 44	40.37	36.12		52.26		
BMI(kg/m^2^)						< 0.001
< 25	31.88	33.44		27.51		
[25, 30)	33.51	34.09		31.88		
≥ 30	34.61	32.47		40.62		
Sex						< 0.001
Male	51.49	53.70		45.28		
Female	48.51	46.30		54.72		
Race/ethnicity						< 0.001
non-Hispanic White	64.70	61.10		74.79		
non-Hispanic Black	11.24	11.75		9.83		
Mexican American	9.27	10.64		5.42		
Other Race/ethnicity	14.79	16.51		9.96		
Marital status						< 0.001
Never married	25.35	26.82		21.23		
Married/living with partner	60.82	62.3		56.67		
Widowed/ divorced	13.83	10.88		22.1		
Poverty income ratio.						0.808
< 1	17.44		17.11		18.36	
[1,3)	33.7		33.89		33.21	
≥ 3	48.86		49.01		48.43	
Education						0.156
Below high school	3.25	3.58		2.35		
High school	30.81	30		33.07		
College or above	65.94	66.42		64.59		
Smoke						< 0.001
Never smoke	58.00	62.67		44.92		
Former smoke	18.79	17.01		23.8		
Current smoke	23.21	20.32		31.28		
Alcohol drinking						0.418
No alcohol use	22.64	23.15		21.2		
Moderate alcohol use	50.13	49.53		51.81		
High alcohol use	27.23	27.31		26.98		
Muscle quality index	3.40 ± 0.02	3.44 ± 0.02		3.29 ± 0.03		< 0.001
Sleep duration (hour)						< 0.001
< 7	55.56	59.35		44.96		
[7, 9)	39.14	35.4		49.59		
≥ 9	5.30	5.25		5.44		
Recreational activity (min/week)						< 0.001
< 150	57.82		55.89		63.23	< 0.001
≥ 150	42.18		44.11		36.77	
Work activity (min/week)						0.129
< 150	63.03		62.07		65.72	
≥ 150	36.97		37.93		34.28	
Sedentary behavior (min/day)						< 0.001
< 480	56.02		57.94		50.60	
≥ 480	43.98		42.06		49.40	

Notes: Mean ± SE for continuous variables and % for categorical variables. NHANES, National Health and Nutrition Examination Survey; BMI, body mass index

**Fig. 2:**
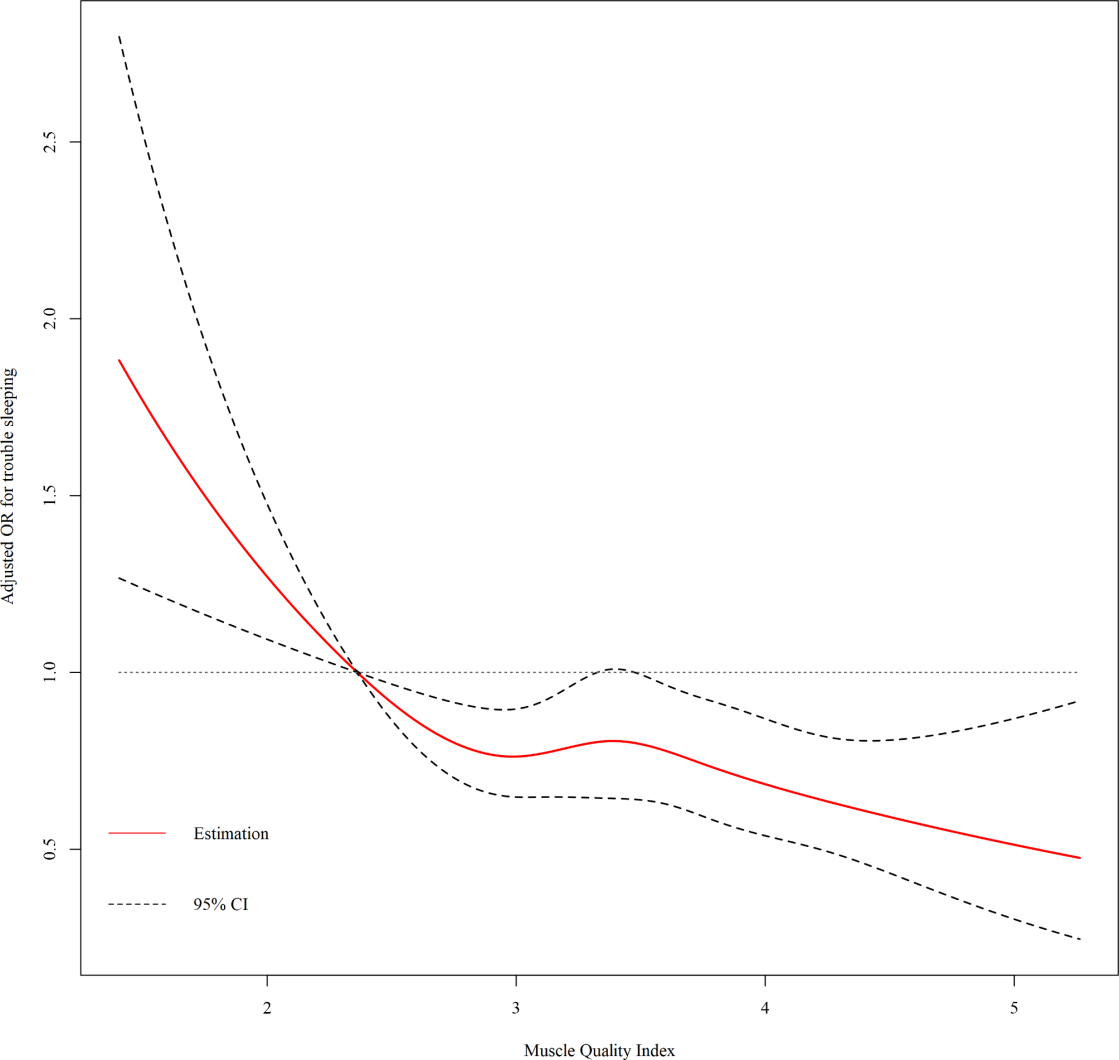
The dose-response relationship between muscle quality index and trouble sleeping. Notes: Age, sex, race, body mass index, marital status, sleep duration, education attainment, poverty income ratio, smoking status, alcohol drinking status, physical activity and sedentary behavior were adjusted in the restricted cubic spline

As shown in Table 2, the MQI level was significantly associated with trouble sleeping. The MQI was negatively associated with the odds ratio of trouble sleeping across all three models (Model 1: OR = 0.680, 95% CI: (0.603,0.768), p < 0.001; Model 2: OR = 0.725, 95% CI: (0.637,0.826), p < 0.001; and Model 3: OR = 0.765, 95% CI: (0.652,0.896), p = 0.011). Considering the significant relation of the MQI with trouble sleeping, a piecewise regression analysis was performed (Table 3). The inflection point of the MQI was 2.362 for trouble sleeping. It was observed that the prevalence of trouble sleeping decreased with increasing MQI until it reached 2.362, after which the odds ratio of trouble sleeping reached a plateau. On the basis of this finding, we performed restricted cubic spline models based on Model 3. From Fig. 2, we can detect that the ORs for the association between MQI and trouble sleeping were decreased with elevated MQI levels. When MQI reached 2.362, the OR was significantly lower than 1.

**Table 2:** Weighted logistic regression results of associations between muscle quality index and trouble sleeping

	OR		95%CI		*P-value*
**Model 1**					
Muscle quality index	0.680		(0.603,0.768)		< 0.001
**Model 2**					
Muscle quality index	0.725		(0.637,0.826)		< 0.001
**Model 3**					
Muscle quality index	0.765		(0.652,0.896)		0.011

Notes: OR = odds ratio, CI = confidence intervals. Model 1, no covariate was adjusted. Model 2, age, sex, race/ethnicity were adjusted. Model 3, age, sex, race, body mass index, marital status, sleep duration, education attainment, poverty income ratio, smoking status, alcohol drinking status, physical activity and sedentary behavior were adjusted

**Table 3:** Threshold effect analysis between muscle quality index and trouble sleeping

Outcome	OR (95% CI)	*P-value*
Two - piecewise linear regression model		
MQI < 2.362	0.384 (0.191, 0.771)	0.007
MQI ≥ 2.362	0.845 (0.730, 0.978)	0.024
Log - likelihood ratio test		0.039

Notes: age, sex, race, body mass index, marital status, sleep duration, education attainment, poverty income ratio, smoking status, alcohol drinking status, physical activity and sedentary behavior were adjusted in the threshold effect analysis

**Fig. 3:**
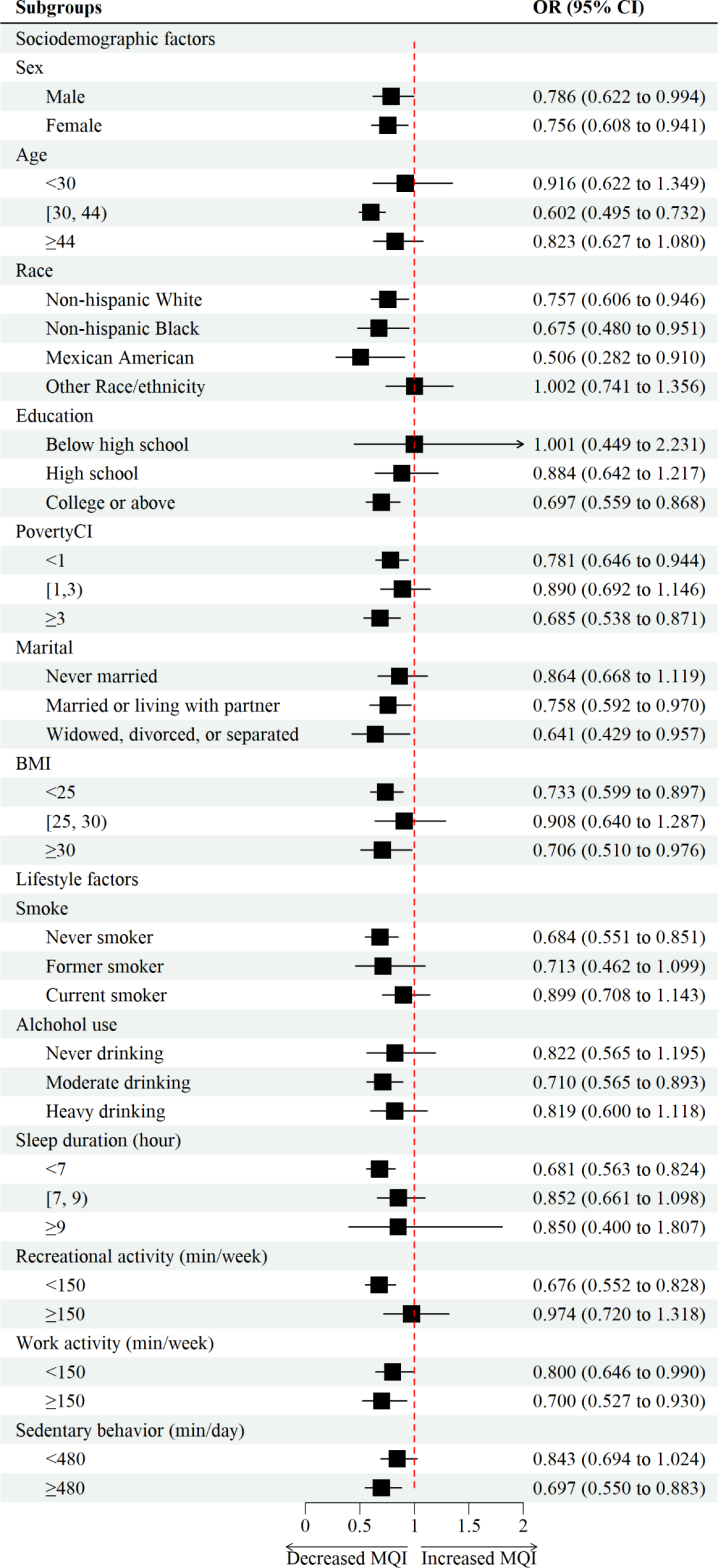
Forest plot of subgroup analysis between muscle quality index and trouble sleeping. Notes: Age, sex, race, body mass index, marital status, sleep duration, education attainment, poverty income ratio, smoking status, alcohol drinking status, physical activity and sedentary behavior were adjusted in the subgroup analysis

In addition, the subgroup analysis (Fig. 3) showed that the negative association between MQI and trouble sleeping was consistent across groups. After stratifying by gender, female participants had a significant decrease in the level of MQI [OR (95% CI) = 0.756(0.608,0.941), p = 0.033]. When stratified by age, the multivariate logistic regression confirmed that participants between 44 and 60 years had a significant decrease [OR (95% CI) = 0.602(0.495,0.732), p < 0.001] in the odds ratio of trouble sleeping, with a gradual decrease as the quality of muscle increased. It was necessary to consider physical activity as an important lifestyle factor. Our results showed that in groups with more work activity, participants had a significant decrease [OR (95% CI) = 0.700(0.527,0.930), p = 0.036] in the prevalence of trouble sleeping with the increment of MQI. However, there was no significant statistical difference in the sufficient recreational activity groups [OR (95% CI) = 0.974(0.720,1.318), p = 0.870]. For groups with more sedentary time, there was also similarly result showing that higher MQI was significantly associated with lower trouble sleeping [OR (95% CI) = 0.697(0.550,0.883), p = 0.015]. Additionally, a stratified analysis showed that these associations were consistent for subgroups with different social-demographic and lifestyle characteristics, which was described in Supplementary Table S1.

## Discussions

To the knowledge of the authors of the current study, this was the first to investigate the role of muscle quality in its relationship with trouble sleeping. We found that MQI was negatively associated with the odds ratio of trouble sleeping. However, the benefits of strong muscle had a threshold, and after which, its effects on reducing trouble sleeping came to a plateau. Subgroup analyses further confirmed these results and discussed the specific relationship in consideration of different influencing factors.

The importance of muscle quality for sleep health and overall well-being has been increasingly recognized. Different from the well-established role of the brain in the sleep process, muscle influence on sleep health is springing up nowadays. Maintaining muscle mass and strength has great potential to prevent sleep disorders including trouble sleeping. When it comes to mechanisms, Scientists recently put forward a revelation that challenges the widely accepted opinion that the brain controls all aspects of sleep and showed that mice with higher expression of the BMAL1 protein in their muscle tissues recovered more quickly from sleep deprivation [19]. Indeed, BMAL1 was a newly detected circadian transcription factor, and circadian factors can govern not only the physiological variables such as sleep–wake cycles, but they also affect the behavior performance [20, 21]. These findings provided evidence that protein in muscles can regulate sleep rhythms. Besides that, classic findings also proved the role of hormones in the relationship between muscle and sleep health. In consistency with sleep rhythm, hormones, represented by testosterone, also followed a rhythm, which troughed at night and started to ascend during sleep and peaked in the morning [22, 23]. Additionally, testosterone was also highly associated with muscle mass and strength [24]. These findings have led us to propose that muscle related factors played important roles in sleep regulation, although these findings warranted further investigations.

Gender and age seemed to be independent factors that affect muscle quality and sleep health. Sex and age difference in muscle quality and trouble sleeping may shed a light on the healthcare utilization, as well as the prevention and treatment of such disorder. Our results detected that in female groups, keeping certain amount of muscle was helpful in reducing the risk of trouble sleeping. Gender differences in sleep problems cannot be ignored as trouble sleeping is generally higher in women groups [25–27], which was similar in our results in Table 1. There is evidence that hormonal steroid, and menopause changes play important roles in this condition [28]. Age is another important factor, the process of aging can make great physical and biological changes in circadian rhythms as well as muscle structure and function [29]. Our subgroup results detected that even in middle aged people from 44 to 60 years, the benefit of muscle quality still existed in preventing trouble sleeping. Previous meta-analysis reported that poor sleep quality was more common in sarcopenia groups [30], thus indicated the importance in maintaining appropriate skeletal muscle mass, strength, and quality.

Lifestyle factors including participating in physical activities have been proved to affect sleep health and muscle quality. A large body of evidence suggested that there were biological and psychosocial mechanisms induced by physical exercise [31–33], which may improve sleep symptoms and quality of life. However, in this current study, we found no significant statistical difference in the sufficient recreational activity groups. One possible explanation for the paradoxical results may be related to the calculation of recreational activity in NHANES. In NHANES, participants self-reported their activity patterns and the frequency (exercise days per week) and duration (exercise times per day). However, the specific exercise type was not reported in detail. One previous research indicated that exercise type and intensity are important influencing factors, and resistance training performed better than aerobic exercise to achieve the benefits of sleep [34]. Another research reported that resistance exercise is an efficacious intervention to improve MQI [35]. This was in agreement with our findings, concerning work activity mostly needed the support of muscle strength, the relationship between MQI and trouble sleeping was significant in sufficient work activity groups. Similar to most alternative and complementary therapy in treating trouble sleeping, the benefits of muscle quality had a dose-response effect, and over the turning point, the effect might tend to weaken. The effect of acute or long-term resistance exercise on muscle quality and its relationship with trouble sleeping warranted further investigation.

The major strength of this current study lay in the large sample size that, on the basis of the complex multi-stage sampling design, which was the representative of the U.S. population. Participants included in our sample represented a wide age range (from 18 to 60 years). Additionally, employing the MQI instead of just muscle mass or grip strength was a stronger indicator of muscle quality. The findings of this research may be of precious value with respect to sleep health concerns. It offered additional insight for health professionals into the viewpoint that trouble sleeping and muscle quality was closely associated. As such, it opened the door for clinical settings in early detection and appropriate management of relative sleep health concerns. Assessments and interventions for preventing or improving trouble sleeping may consider the muscle quality as a new approach and a strong predictor. Non-pharmacologic managements of trouble sleeping were popular choices nowadays and regular physical or resistance exercise has been considered as an effective countermeasure to deal with sleep problems [36–38].

Several potential limitations existed. Firstly, due to the cross-sectional study design, we cannot deduce the causal inference or exclude a bidirectional relationship. Secondly, the measurement of trouble sleeping in this study was based on the self-reported questionnaire, which was subjective rather than objective. It was a pity that the relationship between MQI and severity of trouble sleeping was not assessed in this manuscript. The combined use of Pittsburgh Sleep Quality Index (PSQI), physical activity scanners and polysomnography would be helpful to strengthen investigations in this domain. In addition, it has been previous reported that the prevalence of trouble sleeping and sarcopenia in the elderly population was high [30, 39]. However, the dual-energy X-ray absorptiometry examination in NHANES was not applicable for elders over 60 ages. Issues with regard to trouble sleeping and muscle status in elder groups, especially postmenopausal women those with sarcopenia, have yet to be fully explored. Despite these limitations, this analysis has produced meaningful and interesting findings which can serve as a research basis for further investigations.

## Conclusion

In conclusion, the primary finding of this population-based study was that muscle quality was negatively associated with the odds ratio of trouble sleeping. Also, muscle strength was considered as a vital component of sleep health and there was a threshold effect in the association mentioned above. These findings provided a basis for further studies in this field. There was evidence that MQI can be used as an effective indicator of trouble sleeping. In this regard, health professionals may consider this association as a component of the heath care assessment and the management of people with trouble sleeping complains. Additionally, evidence of this study also encouraged general population to perform regular physical activity, especially resistance activity, to keep certain amount of muscle to improve trouble sleeping.

## Electronic supplementary material

Below is the link to the electronic supplementary material.

Supplementary Material 1

## Data Availability

(ADM) The datasets generated and analyzed for the current study are available in the NHANES repository. These data can be accessed using the following link: https://wwwn.cdc.gov/nchs/nhanes/Default.aspx.
