# Supplementary material for: Muscle quality index is associated with trouble sleeping: a cross-sectional population based study
Source: BMC Public Health. 2023 Mar 14;23:489. doi: 10.1186/s12889-023-15411-6 (PMC10012435; doi:10.1186/s12889-023-15411-6)
Supplement: Supplementary file 1 — Supplementary Material 1 [file 12889_2023_15411_MOESM1_ESM.docx]

Table S1. Weighted logistic regression of stratified results between muscle quality index and odds ratio of trouble sleeping.

|  | Q1 [1.122,2.947] | Q2 (2.947,3.380] | *P-value* | Q3 (3.380,3.811] | *P-value* | Q4 (3.811,5.831] | *P-value* | *P* for trend |
| --- | --- | --- | --- | --- | --- | --- | --- | --- |
| Sociodemographic factors | | | | | | | | |
| Sex |  |  |  |  |  |  |  |  |
| Male | Reference | 0.807(0.517,1.262) | 0.335 | 0.935(0.671,1.302) | 0.682 | 0.535(0.358,0.798) | 0.003 | 0.004 |
| Female | Reference | 0.815(0.637,1.043) | 0.101 | 0.715(0.512,0.999) | 0.049 | 0.695(0.496,0.974) | 0.036 | 0.025 |
| Age |  |  |  |  |  |  |  |  |
| <30 | Reference | 0.664(0.419,1.052) | 0.079 | 1.095(0.680,1.762) | 0.700 | 0.814(0.490,1.353) | 0.414 | 0.921 |
| [30, 44) | Reference | 0.635(0.403,1.000) | 0.050 | 0.691(0.490,0.974) | 0.036 | 0.504(0.357,0.711) | <0.001 | <0.001 |
| ≥44 | Reference | 1.024(0.790,1.326) | 0.855 | 0.833(0.561,1.237) | 0.352 | 0.646(0.465,0.898) | 0.011 | 0.008 |
| Race |  |  |  |  |  |  |  |  |
| Non-hispanic White | Reference | 0.824(0.639,1.063) | 0.131 | 0.824(0.617,1.101) | 0.182 | 0.593(0.472,0.746) | <0.0001 | <0.001 |
| Non-hispanic Black | Reference | 0.734(0.439,1.229) | 0.230 | 0.809(0.488,1.342) | 0.399 | 0.613(0.394,0.956) | 0.032 | 0.075 |
| Mexican American | Reference | 0.706(0.494,1.008) | 0.055 | 0.648(0.456,0.920) | 0.017 | 0.504(0.293,0.867) | 0.015 | 0.006 |
| Other Race/ethnicity | Reference | 0.520(0.278,0.973) | 0.042 | 0.370(0.173,0.787) | 0.012 | 0.374(0.215,0.651) | 0.001 | 0.003 |
| Poverty |  |  |  |  |  |  |  |  |
| <1 | Reference | 1.027(0.683,1.546) | 0.893 | 1.140(0.777,1.673) | 0.489 | 0.556(0.368,0.840) | 0.007 | 0.023 |
| [1,3) | Reference | 0.685(0.495,0.948) | 0.024 | 0.987(0.735,1.325) | 0.929 | 0.526(0.395,0.699) | <0.0001 | 0.002 |
| ≥3 | Reference | 0.775(0.543,1.105) | 0.153 | 0.591(0.401,0.873) | 0.010 | 0.607(0.438,0.840) | 0.004 | 0.001 |
| Marital |  |  |  |  |  |  |  |  |
| Never married | Reference | 0.662(0.378,1.161) | 0.144 | 0.990(0.639,1.533) | 0.962 | 0.572(0.338,0.971) | 0.039 | 0.127 |
| Married or living with partner | Reference | 0.741(0.561,0.978) | 0.035 | 0.694(0.512,0.941) | 0.021 | 0.581(0.437,0.772) | <0.001 | <0.001 |
| Widowed, divorced, or separated | Reference | 1.198(0.728,1.970) | 0.465 | 0.922(0.495,1.719) | 0.793 | 0.589(0.305,1.139) | 0.112 | 0.108 |
| Education |  |  |  |  |  |  |  |  |
| Below high school | Reference | 0.917(0.642,1.311) | 0.624 | 1.065(0.692,1.637) | 0.768 | 0.541(0.380,0.769) | 0.001 | 0.006 |
| High school | Reference | 0.728(0.555,0.953) | 0.023 | 0.675(0.465,0.981) | 0.040 | 0.579(0.422,0.795) | 0.001 | 0.003 |
| College or above | Reference | 1.863(0.686,5.059) | 0.211 | 1.946(0.592,6.401) | 0.259 | 1.455(0.332,6.382) | 0.605 | 0.726 |
| BMI |  |  |  |  |  |  |  |  |
| <25 | Reference | 0.726(0.371,1.423) | 0.339 | 0.935(0.522,1.677) | 0.817 | 0.720(0.395,1.311) | 0.272 | 0.303 |
| [25, 30) | Reference | 1.091(0.627,1.899) | 0.750 | 1.177(0.707,1.959) | 0.518 | 0.800(0.454,1.410) | 0.428 | 0.375 |
| ≥30 | Reference | 0.817(0.593,1.126) | 0.207 | 0.694(0.432,1.115) | 0.126 | 0.536(0.285,1.007) | 0.052 | 0.018 |
| Lifestyle factors | | | | | | | | |
| Smoke |  |  |  |  |  |  |  |  |
| Never smoker | Reference | 0.735(0.559,0.967) | 0.029 | 0.715(0.506,1.012) | 0.058 | 0.447(0.319,0.628) | <0.0001 | <0.0001 |
| Former smoker | Reference | 0.922(0.584,1.457) | 0.719 | 1.044(0.675,1.616) | 0.840 | 0.656(0.462,0.931) | 0.020 | 0.028 |
| Current smoker | Reference | 0.752(0.414,1.367) | 0.337 | 0.650(0.358,1.179) | 0.149 | 0.667(0.372,1.198) | 0.168 | 0.116 |
| Alchohol use |  |  |  |  |  |  |  |  |
| Never drinking | Reference | 1.281(0.837,1.961) | 0.243 | 1.072(0.746,1.540) | 0.698 | 0.618(0.389,0.983) | 0.043 | 0.038 |
| Moderate drinking | Reference | 0.720(0.514,1.009) | 0.056 | 0.682(0.476,0.975) | 0.037 | 0.510(0.368,0.706) | <0.001 | <0.001 |
| Heavy drinking | Reference | 0.600(0.382,0.942) | 0.028 | 0.799(0.511,1.249) | 0.312 | 0.657(0.433,0.996) | 0.048 | 0.17 |
| Sleep duration (hour) |  |  |  |  |  |  |  |  |
| <7 | Reference | 0.661(0.471,0.928) | 0.018 | 0.562(0.396,0.799) | 0.002 | 0.496(0.375,0.657) | <0.0001 | <0.0001 |
| [7, 9) | Reference | 1.098(0.777,1.552) | 0.585 | 1.178(0.861,1.611) | 0.294 | 0.715(0.508,1.007) | 0.054 | 0.104 |
| ≥9 | Reference | 0.580(0.145,2.322) | 0.428 | 0.840(0.310,2.280) | 0.723 | 0.483(0.153,1.531) | 0.207 | 0.28 |
| Recreational activity (min/week) |  |  |  |  |  |  |  |  |
| <150 | Reference | 0.788(0.574,1.080) | 0.133 | 0.757(0.551,1.042) | 0.085 | 0.491(0.373,0.647) | <0.0001 | <0.0001 |
| ≥150 | Reference | 0.907(0.644,1.278) | 0.565 | 0.952(0.666,1.361) | 0.781 | 0.799(0.541,1.178) | 0.247 | 0.324 |
| Work activity (min/week) |  |  |  |  |  |  |  |  |
| <150 | Reference | 0.814(0.616,1.076) | 0.143 | 0.744(0.511,1.082) | 0.117 | 0.635(0.473,0.852) | 0.004 | 0.007 |
| ≥150 | Reference | 0.761(0.514,1.126) | 0.164 | 0.893(0.645,1.237) | 0.484 | 0.503(0.346,0.731) | <0.001 | 0.001 |
| Sedentary behavior (min/day) |  |  |  |  |  |  |  |  |
| <480 | Reference | 0.825(0.657,1.035) | 0.093 | 0.950(0.722,1.249) | 0.702 | 0.664(0.514,0.858) | 0.003 | 0.012 |
| ≥480 | Reference | 0.787(0.578,1.073) | 0.125 | 0.678(0.449,1.022) | 0.063 | 0.515(0.360,0.737) | <0.001 | <0.001 |
